# A quantitative and qualitative examination of the impacts of the COVID-19 pandemic on U.S. veterans with self-reported anxiety and alcohol use disorder

**DOI:** 10.3389/fpsyt.2022.1083212

**Published:** 2023-01-16

**Authors:** Brooke A. Duarte, Megan M. Kelly, Steven D. Shirk, Elizabeth S. Chamberlin, Erin D. Reilly

**Affiliations:** ^1^Department of Psychology, Suffolk University, Boston, MA, United States; ^2^Mental Illness Research, Education, and Clinical Center (MIRECC), VA Bedford Healthcare System, Bedford, MA, United States; ^3^Department of Psychiatry, University of Massachusetts Chan Medical School, Worcester, MA, United States; ^4^Department of Population and Quantitative Health Sciences, University of Massachusetts Medical School, Worcester, MA, United States

**Keywords:** alcohol use disorder, anxiety, veterans, pandemic, functioning

## Abstract

**Introduction:**

The COVID-19 pandemic generated concerns about rising stress and alcohol use, especially in U.S. veterans who experience high rates of anxiety disorders (ADs), alcohol use disorder (AUD), and dual AD+AUD diagnoses. This study investigated differences among these diagnostic groups in a veteran population related to their concern about COVID-19, impacts of COVID-19 on quality of life, and self-reported changes to urge to drink and drinking frequency.

**Methods:**

A nationally administered online survey was given to a sample of U.S. veterans reporting substance use issues during the pandemic. Differences in the level of concern about COVID-19, impacts of COVID-19 on quality of life, and drinking behaviors were examined in those self-reporting AD (*n* = 98), AUD (*n* = 46), or AD+AUD (*n* = 67). Consensual qualitative research was used to analyze an open-ended question about COVID-19’s impact on substance use, health, and quality of life.

**Results:**

Veterans with AD+AUD experienced significant increases in urge to drink and alcohol consumption compared to veterans with AD only. Greater urge and frequency of drinking were associated with greater negative impacts of COVID-19 on quality of life. There were no differences among groups in global negative impact on quality of life or level of COVID-19 concern. However, respondents described specific COVID-19 worries, with qualitative findings revealing that those with AD+AUD reported a disproportionate psychosocial burden due to the pandemic.

**Discussion:**

Special attention in screening and treatment should be given to those with a dual AD+AUD diagnosis who may be experiencing both an increase in alcohol use and psychosocial burden as stress increases due to the pandemic.

## 1. Introduction

As the first waves of the COVID-19 pandemic spread through the United States, stress and anxiety in the general public drastically increased (1–3). Those with pre-existing mental health conditions were of particular concern when considering the impacts of the early stages of the pandemic on functioning (4–6). In times of crisis, having a pre-existing mental health disorder can predispose individuals to an increased likelihood of adverse outcomes such as new or worsening psychological problems, health problems, resource loss, and higher distress (7). Though multiple psychiatric concerns can become more severe during local and global crises, individuals with a pre-existing anxiety disorder (AD) may be especially vulnerable to worsening symptoms.

Anxiety disorders are a heterogeneous group of conditions with varying sources of feared stimuli (i.e., Generalized Anxiety Disorder, Social Anxiety, Panic Disorder, and phobia-related disorders). However, ADs are often grouped together based on their common symptomology for broad-based applications (8, 9). For example, those with ADs experience heightened perceived threat responses characterized by increased physiological arousal, biased threat appraisals, amplified negative emotional states, and behavioral avoidance (10, 11). During the ongoing COVID-19 pandemic, individuals with ADs may be similarly experiencing an overestimation of threat, prolonged fear activation exacerbated by extensive media coverage, anxiety regarding changing public health guidelines, and loss of social support due to increased self-isolation (8).

During crisis and times of elevated stress, there is often increased use of maladaptive coping patterns to avoid, numb, or artificially improve symptoms of anxiety through the use of substances. The first waves of the COVID-19 pandemic brought increased concern about alcohol misuse in particular (12). While many establishments were forced to shut down at the beginning of the pandemic, alcohol sales rose. In March 2020, national alcohol sales in the United States increased by 54% at liquor stores and 262% online compared to 1 year prior (13). Additionally, a May 2020 survey found that U.S. adults were consuming more drinks per day, with a greater proportion of binge drinking than in February 2020 (14). Based on a survey conducted by Grossman et al. (15), reasons for increased alcohol consumption during the pandemic were COVID-19-related stress (45.7%), increased alcohol availability (34.4%), and boredom (30.1%). This is a major concern for public health, given the connection between increased drinking and mortality, rates of addiction, adverse health consequences, and public health harms (16).

U.S. military veterans are at elevated risk for increased anxiety and alcohol use during the pandemic. Prior to the pandemic, U.S. veterans demonstrated higher rates of Alcohol Use Disorder (AUD) than civilians, with over 40% having had a lifetime history (17) compared to under 30% in the general population (18). Likewise, the 12-month rate for any AD is higher among U.S. veterans [33%; (19)] compared to the general population [18.1%; (20)]. This is concerning because individuals with ADs are at risk of experiencing greater COVID-19 stress (8, 9, 21) and report fewer options for coping during the pandemic (22), which may exacerbate drinking. Longitudinal evidence from a nationally representative sample of U.S. veterans suggests that COVID-19-related stress contributed to new and worsening AUD in the U.S. veteran population (23).

Not surprisingly, AUD and ADs are often co-occurring. One theory suggests that individuals with anxiety drink alcohol to cope with symptoms of the disorder, leading to a later onset of AUD, known as the self-medication pathway (24). According to this theory, this comorbidity is problematic because of its mutual maintenance, in which the two disorders influence and maintain each other, leading to decreased global functioning and increased psychosocial difficulties. Those with AD could be experiencing increases in anxiety and stress related to the pandemic and, in turn, using more alcohol to cope. Additionally, the complex clinical presentation of a dual AD+AUD diagnosis may contribute to more COVID-19-related stress and a disproportionate increase in alcohol use as the two disorders exacerbate each other. However, research has yet to examine the impacts of the COVID-19 pandemic on those with a dual AD+AUD diagnosis.

The current study examined COVID-19 experiences and drinking behaviors among U.S. veterans with AD, AUD, or AD+AUD. We hypothesized that those with AD+AUD would experience the greatest increases in urge and frequency of drinking since the beginning of the pandemic and that changes in drinking behavior would be associated with more COVID-19-related stress. We also hypothesized that those with AD+AUD would experience the greatest COVID-19-related concern and negative impacts compared to AD and AUD alone. In addition, we investigated open-ended qualitative data to better understand the self-reported stressors and narrative COVID-19 experiences among these diagnostic groups. A mixed-methods research study includes both quantitative and qualitative data in its collection and analysis. This approach allows for quantitative deductions as well as uncovering more nuanced relationships existing among variables (25). Using a mixed-method design, we expected to see differences among groups such that veterans with AD+AUD would describe more psychosocial concerns (e.g., negative mood impacts, social isolation, and meeting basic needs) compared to either single diagnosis alone.

## 2. Materials and methods

### 2.1. Study design

This study was a planned analysis of data from a nationally representative sample of U.S. veterans managing substance use concerns during the COVID-19 pandemic. The primary study with a full description of the methods is described in Reilly et al. (26). Briefly, the primary study examined the relationship between addiction rates, COVID-19 experiences, and mental and physical functioning among 409 veterans who completed a cross-sectional survey. The current study examined only a subset of those veterans who self-reported an AD and/or AUD diagnosis. This *a priori* decision was theory-driven, given that these groups likely experience a worsening of symptoms during times of crisis (8, 10, 11).

### 2.2. Participants and procedures

Procedures for collecting this data were completed in accordance with a protocol approved by a New England-based VA Healthcare System IRB Committee. The survey was administered using the Qualtrics federal platform, and veterans were identified *via* a Qualtrics panel between 24 November 2020, and 2 February 2021. A Qualtrics panel refers to an internal Qualtrics system where Web-based panel providers have been identified, screened, and utilized by Qualtrics recruiters to supply diverse, quality respondents based on survey inclusion/exclusion criteria. Research has supported the Qualtrics panel recruitment methodology as an effective recruitment strategy (27). This data collection method has evidenced data quality on par with data from conventional data collection methods [e.g., (28)]. Potential panel participants were provided with a link to a description of the study and an eligibility survey on the Qualtrics platform. Informed consent was provided prior to accessing the 30-minute survey.

Eligibility included 18+ years of age, a minimum CAGE-AID score of one (see below for more information), veteran status, including the date of received DD214 (the Certificate of Release or Discharge form), positive endorsement of at least some lifetime alcohol use, and a self-reported AD, AUD, or a dual AD+AUD diagnosis. Therefore, participants were excluded from the current study if they denied lifetime use of alcohol or did not endorse an AD or AUD diagnosis (198 excluded). The final sample for this study included 211 veterans.

### 2.3. Study measures

#### 2.3.1. Demographic variables

Demographic characteristics were self-reported, including age, gender, education, sexual orientation, race, ethnicity, income, armed service branch, and period of service era.

#### 2.3.2. Self-reported diagnostic information

The presence of AD was identified by asking participants to select from a checklist of psychiatric conditions that a doctor or healthcare professional has told them that they have. Those who selected Anxiety Disorder or Panic Disorder from the checklist were included in the AD group. The presence of AUD was identified by asking participants to select from a checklist of medical conditions that a doctor or healthcare professional has told them that they have. Those who selected alcohol use/abuse/addiction were included in the AUD group. Finally, those who selected both Anxiety Disorder or Panic Disorder from the checklist of psychiatric conditions and alcohol use/abuse/addiction from the medical conditions were included in the AD+AUD group.

#### 2.3.3. Addiction measures

At screening, the problematic impact of participants’ alcohol and drug use was assessed using the CAGE Adapted to Include Drugs [CAGE-AID; (29)], a validated four-item measure. An example item is, “Have you ever felt you ought to cut down on your drinking or drug use?” A response of “Yes” is scored as 1 and a response of “No” is scored as 0. A minimum score of 1 was used as a screener to participate in the survey.

A modified version of the Alcohol, Smoking, and Substance Involvement Screening Test [ASSIST; (30)] measured alcohol use frequency, urge, and changes in use during the pandemic. Those who responded “yes” to lifetime use of alcohol completed follow-up questions which were modified to reflect drinking behavior during the pandemic. Modified questions included, “Since the beginning of the COVID-19 pandemic, how often have you drank alcoholic beverages?” and “Since the beginning of COVID-19, how has your desire or urge to drink alcoholic beverages changed?” The study team added items, “How many drinks containing alcohol did you have on a typical day when drinking in the past year?” and “How has your frequency of drinking alcoholic beverages changed during COVID-19?”

#### 2.3.4. COVID-19 measures

The present study evaluated the level of concern about COVID-19, negative impacts on quality of life due to COVID-19, and specific worries related to the pandemic. A subscale score was created using a modified version of the Pain Management Collaboratory Coronavirus Pandemic 5-Item Measure [PMC-5; (31)], which measured the negative impact of COVID-19 on quality of life on a Likert scale of 1 (Improved) to 4 (A Lot Worse). Total scores reflect the average rating across all five items, with higher scores indicating a greater negative impact. The PMC-5 measures finances, emotional health, ability to meet basic needs, physical health, and concentration. For the present study, internal consistency for the PMC-5 was satisfactory (5 items: α = 0.81). Level of COVID-19 concern was measured using one Likert scale item, “How concerned are you about the COVID-19 pandemic?” from 1 (not at all concerned) to 5 (extremely concerned). The Osteoporotic Fractures in Men (MROS) Study COVID-19 Social Impact Questionnaire was used to generate a multiple checkbox item made up of the types of worries assessed in the study conducted by Cawthon et al. (32). In the current study, participants were asked to indicate “Which of the following are worries of yours related to COVID-19, or are more difficult for you now because of the pandemic (Check all that apply)” from a list of worries including getting COVID-19 yourself, someone close to me getting COVID-19, feeling isolated and alone, not getting needed medical or mental health care, meeting basic needs (food, housing, and transportation), finances/income, difficulty meeting conditions of probation or parole, or other.

#### 2.3.5. Open-ended question

Participants were asked to “Please describe anything else related to the impact of coronavirus on you, such as your use of substances/alcohol, mood, relationships, or health.” This open-ended question allowed respondents the opportunity to narratively explain their personal and complex experiences of COVID-19 that multiple-choice items may not have captured. Out of the 211 total participants, 136 (64.5%) provided an answer to this question which were included in qualitative analysis.

### 2.4. Quantitative data analysis

Analyses were conducted using IBM SPSS, version 26 (33). Means, standard deviations, and frequencies were calculated for the full sample. Data were inspected for distribution normality, and no violations were found. Pearson and Spearman Rho correlations were analyzed among study measures to assess relationships among variables. A one-way analysis of variance (ANOVA) for continuous variables (age, CAGE-AID, PMC-5, and COVID-19 concern) and a chi-square test of independence for categorical variables (gender, race, and education) were conducted to examine differences among diagnostic groups. The decision to include demographic variables of age, gender, race, and education was based on previous studies conducted with veterans indicating their impact on mental and physical health outcomes during the COVID-19 pandemic (34–37).

Non-parametric Kruskal-Wallis tests, with a Bonferroni adjusted alpha of 0.017 to correct for multiple tests, were conducted to evaluate changes between diagnostic categories on changes in urge to drinking, drinking frequency, and numbers of drinks consumed when drinking. Additionally, chi-square cross tabulation was used to denote significant differences in drinking behavior between groups in Table 4. Frequencies of specific COVID-19 worries were calculated within each group.

**TABLE 1:** Sample demographics (*N* = 211).

Variable	Frequency (%)	Variable	Frequency (%)
Age	*M* = 49.12 (*SD* = 14.73)	Service branch*	
Gender		Army	106 (50.2%)
Male	153 (73.1%)	Navy	38 (18.0%)
Female	58 (26.9%)	Air force	36 (17.1%)
Race*		Marines	25 (11.8%)
White	192 (91.0%)	National guard	16 (7.6%)
Black/African American	11 (5.2%)	National reserve	6 (2.8%)
Other	4 (1.9%)	Coast guard	3 (1.4%)
American Indian/Alaska Native	3 (1.4%)	Highest education level	
Asian	3 (1.4%)	Less than high school degree	2 (0.9%)
Ethnicity		High school graduate	12 (5.7%)
Not Hispanic/Latino	193 (91.5%)	Some college but no degree	47 (22.3%)
Hispanic/Latino	18 (8.5%)	Associate degree (2-year)	29 (13.7%)
Sexual orientation		Bachelor’s degree (4-year)	58 (27.5%)
Heterosexual (straight)	191 (90.5%)	Master’s degree	53 (25.1%)
Bisexual	11 (5.6%)	Doctoral degree	10 (4.7%)
Homosexual (gay)	7 (3.3%)	Household income	
Prefer not to say	2 (0.9%)	Less than $19,999	19 (9.0%)
Service era*		$20,000–$39,999	38 (18.1%)
September 2001 or later	104 (49.3%)	$40,000–$59,999	28 (13.2%)
August 1990–2001	65 (30.8%)	$60,000–$79,999	22 (10.4%)
May 1975–July 1990	52 (24.6%)	$80,000–$99,999	25 (11.8%)
Vietnam era (1964–1975)	49 (23.2%)	$100,000–$149,999	46 (21.8%)
February 1955–July 1964	4 (1.9%)	$150,000+	33 (15.6%)
November 1941 or earlier	1 (0.5%)		

*Participants could choose multiple categories.

**TABLE 2:** Correlations among study variables.

Variable	M	SD	1	2	3	4
1. CAGEAID	2.73	1.11	–			
2. PMC-5	2.61	0.58	0.15*	–		
3. COVID-19 concern	3.84	1.13	0.12	0.19	–	
4. Greater urge	–	–	0.13	0.24	0.23	–
5. Increased drinking	–	–	0.16*	0.20	0.14*	0.81

*p < 0.05. Correlations between variables 1 and 3 were conducted using Pearson bivariate correlations. Correlations with variables 4 and 5 were conducted using Spearman Rho. CAGEAID, CAGE adapted to include drugs; PMC-5, pain management collaboratory coronavirus pandemic 5-item measure.

**TABLE 3:** Comparisons among diagnostic groups on demographic and study variables of interest.

Variable	AD (*n* = 98)	AUD (*n* = 46)	AD + AUD (*n* = 67)	Statistic	*P*-value
Age	*M* = 51.17 (*SD* = 15.50)^c^	*M* = 54.85 (*SD* = 15.52)^d^	*M* = 42.19 (*SD* = 9.75)^c,d^	13.22^a^	<000
**Gender**					
Male	65	38	50	4.38^b^	0.112
Female	33	8	17		
**Race**					
White	89	40	61	0.63^b^	0.730
Non-white	9	6	6		
**Highest education level**					
Less than bachelor’s degree	45	22	23	2.83^b^	0.243
Bachelor’s degree or above	53	24	44		
**CAGE-AID**	*M* = 2.24^e,f^ (*SD* = 1.11)	*M* = 3.02^e^ (*SD* = 1.04)	*M* = 3.22^f^ (*SD* = 0.83)	20.09^a^	<000
**PMC-5**	*M* = 2.57 (*SD* = 0.53)	*M* = 2.52 (*SD* = 0.59)	*M* = 2.71 (*SD* = 0.64)	2.43^a^	0.174
**COVID-19 concern**	*M* = 3.79 (*SD* = 1.15)	*M* = 3.72 (*SD* = 1.20)	*M* = 4.01 (*SD* = 1.13)	0.86^a^	0.309

^a^*F*, ^b^*X*^2^. AD, anxiety disorder; AUD, alcohol use disorder; AD + AUD, AD and AUD dual diagnosis; CAGEAID, CAGE adapted to include drugs; PMC-5, pain management collaboratory coronavirus pandemic 5-item measure. Significant differences are denoted by a letter corresponding to the between-group analysis (^c–f^).

**TABLE 4:** Drinking behavior among diagnostic groups since the beginning of the pandemic.

	AD (*n* = 98)	AUD (*n* = 46)	AD + AUD (*n* = 67)
Variable	*n* (%)	*n* (%)	*n* (%)
**Drinking frequency**			
Not at all	10 (10.2%)^a^	6 (13.0%)^a^	4 (6.0%)^a^
Once or twice	6 (6.1%)^a^	0 (0.0%)^a^	1 (1.5%)^a^
Monthly	15 (15.3%)^a^	2 (4.3%)^a,b^	2 (3.0%)^b^
Weekly	38 (38.8%)^a^	12 (26.1%)^a^	24 (35.8%)^a^
Daily or mostly daily	29 (29.6%)^a^	26 (56.5%)^b^	36 (53.7%)^b^
# drinks on typical day when drinking*			
1–2	39 (48.1%)^a^	11 (25.6%)^b^	11 (17.5%)^b^
3–4	24 (29.6%)^a^	13 (30.2%)^a^	21 (33.3%)^a^
5–6	10 (12.3%)^a^	7 (16.3%)^a,b^	20 (31.7%)^b^
7–9	3 (3.7%)^a^	6 (14.0%)^a^	6 (9.5%)^a^
10 or more	5 (6.2%)^a^	6 (14.0%)^a^	5 (7.9%)^a^
**Change in desire or urge to drink**			
Lower urge/desire	18 (18.4%)^a^	4 (8.7%)^a^	9 (13.4%)^a^
No change	38 (38.8%)^a^	13 (28.3%)^a,b^	10 (14.9%)^b^
Greater urge/desire	42 (42.9%)^a^	29 (63.0%)^a,b^	48 (71.6%)^b^
**Change in drinking frequency**			
Less drinking	16 (16.3%)^a^	5 (10.9%)^a^	15 (22.4%)^a^
No change	41 (41.8%)^a^	12 (26.1%)^a^	6 (9.0%)^b^
More drinking	41 (41.8%)^a^	29 (63.0%)^a,b^	46 (68.7%)^b^

*Question added after the start of data collection. Responses reflect part of the full sample. AD, anxiety disorder; AUD, alcohol use disorder; AD + AUD, dual diagnosis of anxiety disorder and alcohol use disorder. Each subscript letter denotes a subset of diagnostic categories whose column proportions do not differ significantly from each other based on chi-square tests.

### 2.5. Qualitative coding methodology

The researchers utilized a modified consensual qualitative research approach to analyze data from the open-ended question about COVID-19 impact. Two raters independently examined 20% of narratives to create an initial domain list of common ideas. The researchers followed an abbreviated consensus procedure to discuss their domain formation, based on the procedure described by Hill et al. (38). Researchers coded independently and then met to discuss their domains [see (39)]. Since data were not interview transcripts but a single open-ended item, the researchers abbreviated Hill et al.’s procedure to report on domain themes and frequency classification [see (40)]. The researchers dialogued until consensus was achieved for a domain coding structure. The researchers used this structure to recode the 20% of participant narratives before checking inter-rater reliability (90%). A third researcher operated as an auditor for agreeance on the coding and domain formulation. After the three researchers dialogued and reached consensus regarding a finalized domain coding structure, all narratives in the sample were coded by the two original raters. The two researchers met again to check inter-rater reliability of the full sample coding (99%) and came to consensus on differences. Finally, the three researchers met to group the coded similar domains into thematic schemes. Differences and similarities among thematic scheme and subdomain frequencies were then examined among the three diagnostic groups.

Per reporting guidelines established for consensual qualitative research (38, 41), themes discussed are classified as general if they apply to nearly all cases, typical if they apply to about half or more of the cases, variant if they apply to slightly less than half of cases, or rare if they apply to less than 25% of cases. Responses could be double coded. Frequent codes within the themes were further discussed if they were applied at least twice as frequently for one diagnostic group compared to the other, indicating substantial differences in COVID-19-related impacts and responses between groups.

## 3. Results

### 3.1. Respondents

Table 1 displays detailed information regarding respondent characteristics. The mean age of the sample (*N* = 211) was 49.12 years (*SD* = 14.73), with most participants male (73.1%). Participants could identify with multiple racial categories, and 91.0% identified as White/Caucasian, 5.2% as Black/African American, 1.4% as American Indian or Alaska Native, 1.4% as Asian, and 1.9% as other. Additionally, 8.5% of participants identified as Hispanic, and 57.3% had a bachelor’s degree or higher.

### 3.2. Groupwise comparisons for demographic and COVID-19 variables

Pearson correlations between variables of interest are reported in Table 2, and groupwise comparisons of demographics and study variables of interest using chi-square tests of independence and ANOVA are reported in Table 3. Chi-square tests of independence indicated no significant differences between diagnostic groups on gender (*p* = 0.112), education level (*p* = 0.243), or race (*p* = 0.730). ANOVAs indicated that the average age significantly differed between groups [*F*_(2, 209)_ = 13.33, *p* < 0.000] such that the AD group (*M* = 51.17, *SD* = 15.50) and AUD group (*M* = 54.85, *SD* = 15.52) were on average older than the AD+AUD group (*M* = 42.19, *SD* = 9.75). Given this finding, additional preliminary tests were run to explore a potential effect for age in substance use outcome analyses. There were no significant differences among diagnostic groups on the PMC-5 total score (*p* = 0.174) or COVID-19 concern (*p* = 0.309).

### 3.3. Drinking behavior

Diagnostic groups significantly differed on CAGE-AID scores measuring the problematic impact of drinking, *F*_(2, 209)_ = 20.09, *p* < 0.000. *Post-hoc* comparisons using a Bonferroni correction indicated that those with AD (*M* = 2.24, *SD* = 1.11) scored lower on the CAGE-AID compared to those with AUD (*M* = 3.02, *SD* = 1.04) and AD+AUD (*M* = 3.22, *SD* = 0.83). The AUD group and AD+AUD group did not significantly differ on the CAGE-AID.

Descriptive statistics for alcohol consumption, changes in desire or urge to drink, and changes in drinking frequency since the beginning of the COVID-19 pandemic among each diagnostic group are reported in Table 4. Among the AD group (*n* = 98), 89.8% (88/98) reported drinking since the beginning of the pandemic, with almost one-third (29.6%, 29/98) reporting daily or mostly daily drinking. In the AUD group (*n* = 46), 86.9% (40/46) reported drinking since the beginning of the pandemic, and over half (56.5%, 26/46) reported drinking daily or mostly daily. In the AD+AUD group (*n* = 67), 94.0% (63/67) reported drinking since the beginning of the pandemic, and over half (53.7%, 36/67) reported daily or mostly daily drinking. Greater urge to drink was reported by 42.9% (42/98) of those in the AD group, 63% (29/46) in the AUD group, and 71.6% (48/67) in the AD+AUD group. Similarly, greater drinking frequency since the beginning of the pandemic was reported by 41.8% (41/98) in the AD group, 63% (29/46) in the AUD group, and 68.7% (46/67) in the AD+AUD group. There was no main effect for age as a covariate for urge to drink (*p* = 0.10) or frequency of drinking (*p* = 0.24), but non-parametric Kruskal–Wallis tests revealed significant group differences across diagnostic groups (AD, AUD, and AD+AUD) on both changes in urges to drink, H (2) = 14.82, *p* < 0.001 and changes in frequency of drinking, H (2) = 18.11, *p* < 0.001. *Post-hoc* tests revealed a significant difference between the AD and AD+AUD groups on changes in urge to drink alcohol (*Z* = −3.19, *p* < 0.001), with Veterans with the dual AD+AUD diagnosis reporting significantly greater increases in urge or desire to drink compared to veterans with only AD. Similarly, there was a difference between the AD and AD+AUD groups on changes in urge to drink alcohol (*Z* = −3.19, *p* = 0.02), with Veterans with the dual AD+AUD diagnosis reporting greater increases in urge or desire to drink compared to veterans with only AD, although this association did not meet the Bonferroni adjusted alpha of 0.017.

Regarding the number of drinks consumed on a typical day when drinking, about half of the AD group who provided a response (48.1%, 39/81) reported only drinking 1–2 drinks on a typical day when drinking. In contrast, 25.6% (11/43) of those with AUD who provided a response reported 1–2 drinks per day when drinking and the other 74.5% (32/43) reported 3–10 or more drinks. The AD+AUD group reported the most drinks in a typical day; of those who provided a response, only 17.5% (11/63) reported 1–2 drinks and 82.4% (52/63) reported 3–10 or more drinks. Non-parametric Kruskal–Wallis tests revealed no significant effect of age on number of drinks consumed per day (*p* = 0.24), but there were significant group differences across diagnostic groups (AD, AUD, and AD+AUD) on reported number of drinks in a typical drinking day, H (2) = 17.30, *p* < 0.001. *Post-hoc* tests revealed a significant difference between the AD and AUD groups, such that those in the AUD group reported consuming significantly more alcoholic beverages than those in the AD group when drinking (*Z* = −2.93, *p* = 0.003). In addition, there was a significant difference between the AD and AD+AUD groups (*Z* = −3.92, *p* < 0.001), with the AD+AUD group reporting higher rates of drinking.

### 3.4. Specific COVID-19 worries

**FIGURE 1:**
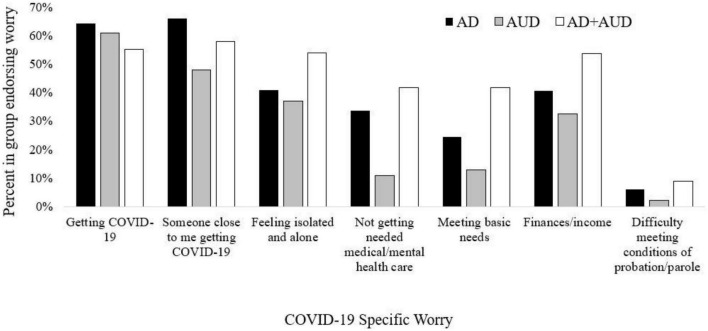
COVID-19 specific worries by diagnostic group. Specific COVID-19 worries are displayed by the percentage of people who endorsed them within each diagnostic group. AD, anxiety disorder; AUD, alcohol use disorder; AD + AUD, dual diagnosis of anxiety disorder and alcohol use disorder.

Figure 1 displays the specific COVID-19 worries reported by each diagnostic group. Worries about getting infected with COVID-19 were similar between those with AD (64.3%, 63/98) and AUD (60.9%, 28/46). This worry was slightly lower for those with both AD+AUD (55.2%, 37/67). The group most worried about someone close getting COVID-19 was those with AD (66.3%, 65/98), followed by AD+AUD (58.2%, 39/67), and then AUD (47.8%, 22/46). Worries about feeling isolated and alone a lot of the time were reported most frequently by those with AD+AUD (53.7%, 36/67), followed by AD (40.8%, 40/98) and AUD (37.0%, 17/46). There was a similar pattern in worries about inaccessibility to medical or mental health care as well as meeting basic needs, such that the AD+AUD group reported the most worry about these needs (41.8%, 28/67 for both categories), followed by AD (33.7%, 33/98 and 24.5%, 24/98, respectively) and AUD (10.9%, 5/46 and 13.0%, 6/46, respectively). Similarly, worries about finances and/or income were reported most often by the AD+AUD group (53.7%, 36/67), followed by AD (40.8%, 40/98), and AUD (32.6%, 15/46). Finally, having difficulty meeting conditions of probation or parole was reported by 9.0% (6/67) of those with AD+AUD, 6.1% (6/98) with AD, and 2.2% (1/46) with AUD.

### 3.5. Qualitative results

Table 5 reports the detailed qualitative results and example quotes. Open-ended text responses analyzed were provided from 54 out of 98 total participants in the AD group (55.1%), 32 out of 46 in the AUD group (69.6%), and 50 out of 67 in the AD+AUD group (74.6%). Codes within each theme are presented below by the diagnostic group. For those with AD, a variant theme of increased psychological distress applied to 44.4% of cases. A variant theme of reduced access to recreational options (24.1%) also emerged, with specific codes related to reduced ability to engage in activities due to restrictions (61.5%), travel interference (30.8%), and boredom (7.7%). For those with AUD, increased psychological distress was a variant theme (43.8%). The theme of negative social impact was variant (28.1%), including codes of loneliness/isolation (77.7%) and relationship impacts (22.2%). Reduced access to recreational options was a variant theme (25.0%), including codes of reduced ability to engage in activities due to restrictions (75.0%), travel interference (12.5%), and boredom (12.5%). For those with AD+AUD, the theme of increased psychological distress was typical (60.0%). Negative social impact was a variant theme (32.0%), including codes of loneliness/isolation (81.3%) and relationship impacts (18.8%).

**TABLE 5:** Qualitative analysis and example quotes of negative COVID-19 impacts among diagnostic groups.

	AD (*n* = 54)		AUD (*n* = 32)		AD + AUD (*n* = 50)			
Thematic scheme	Codes	Classification	*n (%)	Classification	*n (%)	Classification	*n (%)	Quotes
Increased psychological distress		Variant	24 (44.4%)	Variant	14 (43.8%)	Typical	30 (60.0%)	
	Negative mood impact		9 (37.5%)		1 (7.1%)		9 (30%)	“I’m depressed and lonely. I’m stir crazy. Bored. Sad. Worried. And broke.” (AD + AUD)
	Increased anxiety/stress		9 (37.5%)		1 (7.1%)		7 (23.3%)	“My cravings for alcohol increased and I became more anxious.” (AD+AUD)
	Increased substance use		6 (25.0%)		12 (85.7%)		14 (46.7%)	Just bad (because) one is looking for something to use to pass time. “It sucks.” (AUD)
Negative social impact		Rare	10 (18.5%)	Variant	9 (28.1%)	Variant	16 (32.0%)	
	Loneliness/isolation		7 (70%)		7 (77.7%)		13 (81.3%)	“I feel so lonely so I get drunk or use substances to cheer myself up.” (AD+AUD)
	Relationship impact		3 (30%)		2 (22.2%)		3 (18.8%)	“Poor relationships and I feel angry all the time.” (AUD)
Reduced access to recreational options		Variant	13 (24.1%)	Variant	8 (25%)	Rare	10 (20%)	
	Travel interference		4 (30.8%)		1 (12.5%)		0 (0.0%)	“It is a little depression going through a mid west winter without a trip or outing to energize me. I usually have a trip planned but nothing this year.” (AD)
	Boredom		1 (7.7%)		1 (12.5%)		3 (33.3%)	“I never drank before, but now I do more often out of boredom.” (AD)
	Reduced ability to engage in activities due to restrictions		8 (61.5%)		6 (75%)		7 (70.0%)	“Can’t travel, can’t play cards with friends, can’t go outside anymore, can’t eat out at restaurants.” (AD)
Difficulties making and/or maintaining a living		Rare	5 (9.3%)	Rare	4 (12.5%)	Rare	2 (4.0%)	
	Employment/job issues		3 (60%)		2 (50%)		1 (50%)	“I’ve been drinking more and my hours at work were cut.” (AUD)
	Financial/monetary issues		2 (40%)		2 (50%)		1 (50%)	“It’s just very difficult to make ends meet and I can not find a job.” (AUD)
Fear of COVID-19 infection		Rare	4 (7.4%)	Rare	1 (2.9%)	Rare	2 (4.0%)	“Just stressed about getting it and dying.” (AD)
General negative impacts		Rare	4 (7.4%)	Rare	3 (9.3%)	Rare	3 (6.0%)	“What am I supposed to say. This Corona thing made everything even worse.” (AD+AUD)
Physical/sleep/health impacts		Rare	3 (5.6%)	Rare	1 (2.9%)	Rare	5 (10.0%)	“Even though I have free time, I don’t feel motivated to anything like study, improve my mind, or exercise.” (AD)

*Frequency of thematic schemes was calculated based on total responses in diagnostic group. Frequency of codes was calculated based on total responses in thematic scheme.

When comparing across diagnostic groups within the theme of increased psychological distress, negative mood impacts were reported around five times more frequently in the AD group (37.5%) and four times more often in the AD+AUD group (30.0%) compared to the AUD group (7.1%). Increased anxiety/stress was reported around five times more frequently in the AD group (37.5%) and three times more frequently in the AD+AUD group (23.3%) compared to the AUD group (7.1%). Increased substance use was reported three times more frequently in the AUD group (85.7%) and around two times more frequently in the AD+AUD group (46.7%) compared to the AD group (25.0%).

## 4. Discussion

The primary aim of this study was to examine the COVID-19 experiences of U.S. veterans with AD, AUD, or a dual AD+AUD diagnosis. Specifically, we were interested in (1) how drinking behaviors have changed during the pandemic and (2) if COVID-19 stressors differed among these groups. Descriptively, results indicated that veterans with AD+AUD reported a greater urge to drink and frequency of drinking as well as increases in the number of drinks consumed while drinking during the pandemic compared to the AD only group. A dual AD+AUD diagnosis is often associated with greater symptom severity, impairment, and suicidality compared to having only one disorder (42). Thus, almost 3 out of 4 veterans with AD+AUD in our sample reporting increased drinking indicates the potential for a major public health concern.

However, this issue was not solely associated with veterans reporting an AD+AUD diagnosis; all diagnostic groups reported increased alcohol consumption. This finding is not surprising; prior studies have shown that as stress, boredom, and social isolation increase in the context of disaster, stress-induced alcohol consumption is likely to follow, regardless of mental health status (12, 43). Notably, even those in the AD group without a diagnosis of AUD still reported frequent drinking and increases in the amount of drinking and desire to drink. Additionally, within the sample, a greater urge to drink and increased frequency of drinking were associated with more negative impacts on quality of life due to the pandemic as well as increased concern about COVID-19. These findings add to the considerable amount of research showing that alcohol consumption is used as a coping strategy during public health and economic crises (44–46) and support the self-medication hypothesis. This highlights the importance of screening and assessment of problematic alcohol use for patients even without a known or reported AUD diagnosis, especially for those who may be more vulnerable to stress-induced drinking, such as those with an AD.

Despite reported increases in drinking across groups, there were no meaningful differences among those with AD, AUD, or AD+AUD on the negative impact of COVID-19 on quality of life (PMC-5) or level of concern about COVID-19 (one item Likert scale item). While prior studies found that those with AD experienced elevated COVID-19 stress compared to other clinical groups (8, 21), there is longitudinal evidence suggesting a decrease in symptoms over time may be stronger in those with anxiety (9), potentially due to an increased tendency to initially overestimate threat. Given that our data was collected after the first wave of the pandemic, initial elevations in COVID-19-related stress among those with AD+AUD may have already leveled out, making differences between groups indistinguishable. General stress level, regardless of having anxiety or not, may be a better predictor of increased drinking during the pandemic. Alternatively, those with AUD may be experiencing a similar level of concern about the pandemic as those with anxiety. This anxiety is not without merit as alcohol use can reduce immunity and increase the risk of infection (47) and severity of COVID-19 illness (48).

Although the diagnostic groups may be experiencing similar global negative impacts and concerns about the pandemic, a closer look at specific COVID-19 worries and narrative responses from veterans provided a more nuanced understanding of differences in pandemic experiences. Those with AD+AUD more frequently endorsed worries related to feeling isolated and alone, not getting needed medical or mental health care, meeting basic needs, finances/income, and meeting conditions of probation/parole. In comparison, those with AD reported more worries about themselves or someone close to them getting COVID-19. These findings illuminate the real-world pandemic impact on functioning for those living with AD+AUD such that while these individuals may have comparable global impacts on quality of life as other diagnostic groups, there seems to be an added burden on psychosocial needs, such as loneliness, difficulty maintaining health care needs, and stress on finances and meeting basic needs.

Our qualitative results support this notion as well, pointing to a double disadvantage of psychological distress and increased drinking in the AD+AUD group, which may explain their added psychosocial burdens. The theme of increased psychological distress appeared most frequently in all groups, however, frequency of codes within the theme, or what factors are actually contributing to psychological distress, differed. For example, the theme of psychological distress in the AUD group consists almost primarily of concerns about increased substance use, with minimal concerns about negative mood impacts or increased anxiety/stress. Conversely, negative mood impacts and increased anxiety/stress codes were more common in the AD and AD+AUD groups, indicating that having an anxiety diagnosis may contribute to mental health concerns. Further, the AD+AUD group displayed an additional public health issue, with increased substance use reported around two times more frequently than the AD group. The AD+AUD group frequently described concerns about increased substance use and poorer mental health, whereas the other two groups mainly described experiencing one or the other. Factors associated with increased drinking during the pandemic in the AUD group may be less related to self-medication, and more related to change in routine, decreased access to sobriety resources, and less activity of the reward pathways associated with addiction (49). However, increases in negative mood and anxiety symptoms within the AD and AD+AUD groups are likely contributing to the use of alcohol as a coping strategy to alleviate distress during the pandemic, with this alcohol-consumption impact exacerbated in individuals with a dual diagnosis.

The proportion of COVID-19-related worries and concerns reported within groups in the current study were included largely for exploratory purposes; however, they provided a more contextual understanding of the COVID-19 experiences within this community sample of Veterans. Specific areas of worries have been shown to be important in understanding the coping and stress response of many individuals during COVID-19 (50). Therefore, future research and secondary analyses of COVID-19 data should explore this issue more fully. Differences in particular stressors, and their impact on mental health and substance use, might also differ for people from different sub-populations (e.g., gender, underlying health conditions, and mental health diagnoses). Understanding the differential impacts of specific COVID-19 stressors can inform intervention efforts in the ever-evolving public health situation.

### 4.1. Limitations

This study has several limitations. First, generalizability to all veterans is limited, as everyone in the sample reported at least some problematic experiences of substance use in the past 12 months (measured by the CAGE-AID) to be included in the study. Therefore, all participants had experienced some recent addiction issues with possible impact of additional substances. However, the current paper focused solely on alcohol which was in line with the theoretical framework presented as well as that being the most commonly reported substance used in the primary study (26). Generalizability may also be limited with a sample of 91% White/Caucasian veterans, which is higher than the national estimate of non-Hispanic White veterans [80%; (51)]. Additionally, mental health diagnoses being self-reported and not independently rated by a clinician could threaten validity of the diagnostic groups compared in the current analyses. However, prior studies support the use of self-reported diagnoses as adequate indicators of mental health status (52, 53). Another limitation is the small sample size of diagnostic groups which limits the ability to distinguish differences between groups and generalize findings. The current study utilized modified versions of the ASSIST, PMC-5, and MROS. Although the authors believe the modifications were consistent with the original use of each measure, results drawn from modified measures that have not been validated should be interpreted with caution. Finally, a panel-recruited, web-based survey methodology may be susceptible to fraudulent and biased responses. However, standardized quality control reviews and data inclusion screening procedures were implemented to minimize this concern.

## 5. Conclusion

Screening and treatment for rising alcohol use are needed as pandemic stress exacerbates drinking among veterans. Particular attention should be given to those with a dual AD+AUD diagnosis who may be experiencing both an increase in alcohol use and a disproportionate psychosocial burden as stress increases due to the pandemic. Mitigating these concerns will continue to be an issue during the ongoing pandemic, and greater efforts to address them will be vital to veterans and civilians.

## Data availability statement

The original contributions presented in this study are included in the article, further inquiries can be directed to the corresponding author.

## Ethics statement

The studies involving human participants were reviewed and approved by the VA Bedford Healthcare System IRB Committee. The patients/participants provided their written informed consent to participate in this study.

## Author contributions

ER conceptualized the study. BD, ER, and MK completed the qualitative analysis. BD wrote the manuscript with the help of all coauthors. All authors contributed to the study design and implementation, and reviewed and agreed with the final manuscript prior to submission.
